# Mapping of QTL for Resistance against the Crucifer Specialist Herbivore *Pieris brassicae* in a New Arabidopsis Inbred Line Population, Da(1)-12×Ei-2

**DOI:** 10.1371/journal.pone.0000578

**Published:** 2007-06-27

**Authors:** Marina Pfalz, Heiko Vogel, Thomas Mitchell-Olds, Juergen Kroymann

**Affiliations:** 1 Department of Genetics & Evolution, Max Planck Institute for Chemical Ecology, Jena, Germany; 2 Department of Entomology, Max Planck Institute for Chemical Ecology, Jena, Germany; 3 Department of Biology, Duke University, Durham, North Carolina, United States of America; Cairo University, Egypt

## Abstract

**Background:**

In *Arabidopsis thaliana* and other crucifers, the glucosinolate-myrosinase system contributes to resistance against herbivory by generalist insects. As yet, it is unclear how crucifers defend themselves against crucifer-specialist insect herbivores.

**Methodology/Principal Findings:**

We analyzed natural variation for resistance against two crucifer specialist lepidopteran herbivores, *Pieris brassicae* and *Plutella xylostella*, among *Arabidopsis thaliana* accessions and in a new Arabidopsis recombinant inbred line (RIL) population generated from the parental accessions Da(1)-12 and Ei-2. This RIL population consists of 201 individual F_8_ lines genotyped with 84 PCR-based markers. We identified six QTL for resistance against *Pieris* herbivory, but found only one weak QTL for *Plutella* resistance. To elucidate potential factors causing these resistance QTL, we investigated leaf hair (trichome) density, glucosinolates and myrosinase activity, traits known to influence herbivory by generalist insects. We identified several previously unknown QTL for these traits, some of which display a complex pattern of epistatic interactions.

**Conclusions/Significance:**

Although some trichome, glucosinolate or myrosinase QTL co-localize with *Pieris* QTL, none of these traits explained the resistance QTL convincingly, indicating that resistance against specialist insect herbivores is influenced by other traits than resistance against generalists.

## Introduction

*Arabidopsis thaliana* recombinant inbred lines (RILs) have been widely used for mapping of quantitative trait loci (QTL) (reviewed in [1]). Taking advantage of RILs derived from crosses between the accessions Columbia (Col) and Landsberg *erecta* (L*er*) [2], and between L*er* and Cape Verdi Islands (Cvi) [3], several insect resistance QTL have been mapped and, subsequently, several were cloned and characterized [4]–[8]. In most cases, these studies involved lepidopteran species with a broad host range (generalists) such as *Spodoptera exigua* or *Trichoplusia ni*, and found that generalist insects were sensitive towards glucosinolate-based defenses. Glucosinolates (β-thioglucoside–N-hydroxysulfates) are amino acid-derived secondary plant metabolites that can be hydrolyzed by myrosinases, enzymes with β-thioglucoside glucohydrolase activity [9]–[11]. In *Arabidopsis thaliana*, three major classes of glucosinolates are known: aliphatic glucosinolates derived from chain-extended methionine homologues, indole glucosinolates derived from tryptophan, and benzyl glucosinolates originating from a phenylalanine precursor [12]. Two major loci, *AOP* [13], [14] and *MAM* [7], [15]–[17], and several minor loci [18] control composition and quantity of aliphatic glucosinolates. Methylthioalkylmalate synthases encoded at the *MAM* locus determine the side chain length of the methionine-derived precursors, while 2-oxoglutarate-dependent dioxygenases encoded at *AOP* modify the side chain structure. In intact plant tissue, glucosinolates and myrosinases are localized in separate cell types [19]–[22]. Upon tissue disruption, myrosinase-catalyzed glucosinolate hydrolysis results in the formation of bioactive products, including isothiocyanates, nitriles, thiocyanates and others [23]. The types of breakdown products formed depend on the glucosinolate structure, as well as on myrosinase-associated or –binding proteins that can direct the formation of breakdown products towards nitriles or isothiocyanates [5], [8]. Typically, plant damage caused by generalist insect herbivores is negatively correlated with increasing glucosinolate concentration or myrosinase activity, and resistance QTL co-localize with glucosinolate biosynthesis or hydrolysis QTL, providing evidence for a major role of the glucosinolate-myrosinase system in the defense of cruciferous plants against generalist insect herbivores [5]–[9], [24].

Entirely unclear, however, is how cruciferous plants defend themselves against specialist insect herbivores. Several counteradaptations have been identified in crucifer specialist lepidopterans that render the glucosinolate-myrosinase system ineffective. *Plutella xylostella* (diamondback moth) larvae express a glucosinolate sulfatase in their gut that removes the sulfate moiety from glucosinolates, thereby preventing myrosinase-catalyzed hydrolysis and formation of toxic breakdown products [25]. *Pieris rapae* (cabbage white butterfly) possesses a nitrile-specifier protein (NSP) that redirects glucosinolate hydrolysis towards the formation of nitriles instead of highly toxic isothiocyanates when plant tissue is ingested by *Pieris* larvae [26]. Nonetheless, Arabidopsis accessions vary for resistance against specialist insect herbivores. In this paper, we analyze quantitative genetic variation for resistance against two crucifer specialist lepidopteran herbivores, *Pieris brassicae* and *Plutella xylostella*, among Arabidopsis accessions and in a new Arabidopsis recombinant inbred line, Da(1)-12×Ei-2, and we investigate whether variation in glucosinolate profiles, myrosinase activity or trichomes contributes to variation in resistance.

## Results

### Natural Variation for Resistance against Crucifer Specialist Insect Herbivores among Arabidopsis Accessions

**Figure 1:**
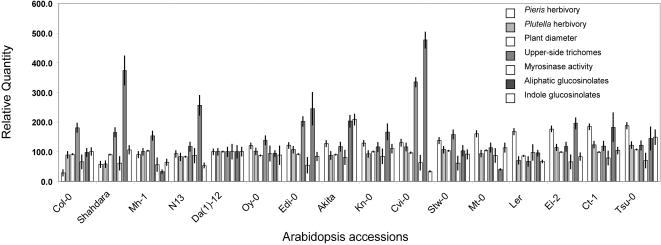
Natural variation among 16 Arabidopsis accessions for insect resistance, growth and defense-related traits. Accessions are ranked according to increasing susceptibility to herbivory by *Pieris brassicae* larvae. Shown are least squares means and standard errors (vertical bars) for *Pieris brassicae* herbivory, *Plutella xylostella* herbivory, plant diameter, leaf upper-side trichomes, myrosinase activity, aliphatic and indole glucosinolates. Values for Da(1)-12 are set as 100.

We analyzed 16 Arabidopsis accessions for natural variation in resistance against two crucifer specialist insects, *Pieris brassicae* and *Plutella xylostella*. We found substantial variation for resistance against *P. brassicae* (*F* = 20.31, *df* = 15, *N* = 973, *P*<0.00001), with Col-0 being the most resistant and Tsu-0 the most susceptible accession (**Figure 1**). Variation for resistance against *P. xylostella* was less pronounced, but nonetheless statistically significant (*F* = 2.38, *df* = 15, *N* = 912, *P*<0.005). Resistance to *P. brassicae* and *P. xylostella* was positively correlated (*r* = 0.55, *df* = 14, *P*<0.05), suggesting that some determinants of plant resistance affect both specialists similarly.

We also analyzed natural genetic variation for trichome density, glucosinolate content and myrosinase activity, traits known to influence resistance against generalist insect herbivores. As expected, these traits varied among Arabidopsis accessions (**Figure 1**). However, no single trait alone could explain the observed variation in resistance to *P. xylostella* or *P. brassicae* among Arabidopsis accessions. We therefore chose to analyze quantitative variation for resistance and defense-related traits in a new Arabidopsis recombinant inbred line population, derived from a cross between the parental accessions Da(1)-12 and Ei-2 [5]. Although these lines did not represent the extreme phenotypes in the distribution of resistance against *P. brassicae*, they provided a variety of advantages regarding the composition of alleles at glucosinolate biosynthesis and hydrolysis loci compared to ‘standard’ mapping populations such as Col×L*er* (2) or L*er*×Cvi [3]. Leaves of Da(1)-12 and Ei-2 synthesize aliphatic and indole glucosinolates. Both lines accumulate similar quantities of glucosinolates in their leaf tissue (**Figure 1**). Furthermore, in both lines the predominant aliphatic glucosinolates are derived from a homo-methionine precursor, indicating the presence of an intact *MAM2* gene and the absence of a functional *MAM1* gene in the *MAM* gene cluster [7], [27]. However, Da(1)-12 and Ei-2 differ in their alleles at the *AOP* locus [14]. Da(1)-12 possesses an *OHP* allele at *AOP*, while Ei-2 carries an *ALK* allele. Therefore, Da(1)-12 produces mainly 3-hydroxypropyl and 3-methylsulfinyl glucosinolates, and Ei-2 accumulates allyl glucosinolate. Finally, during glucosinolate hydrolysis, Da(1)-12 produces isothiocyanates whereas Ei-2 generates predominantly nitriles [5]. This combination of alleles at glucosinolate biosynthesis and hydrolysis loci helps reduce complexity in the investigation of potential impact of the glucosinolate-myrosinase system [9] on herbivory. It avoids epistatic interactions between known major biosynthesis loci, *AOP* and *MAM*, [12] while allowing analysis of potential effects of interactions between glucosinolate biosynthesis and hydrolysis loci on crucifer specialists. In addition, growth rates of both Da(1)-12 and Ei-2 were nearly identical (**Figure 1**), improving the accuracy of estimating the quantity of tissue removal during herbivory.

### RIL Genotyping

**Figure 2:**
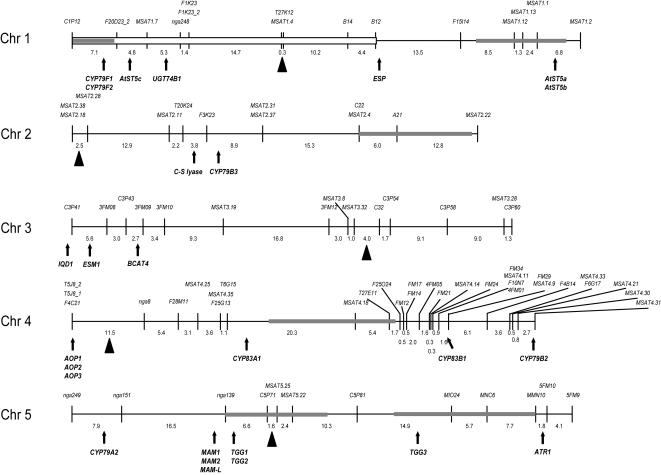
Genetic map of Da(1)-12×Ei-2 recombinant inbred lines. Shown are markers and genetic distances between adjacent markers along the chromosomes. Triangles indicate the approximate location of centromeres. The white bar for chromosome 1 indicates a region with marker distortion. Grey bars indicate 2-LOD support intervals for *Pieris* resistance QTL. Known genes involved in glucosinolate biosynthesis, hydrolysis, and gene regulation are shown below chromosomes; explanations, AGI numbers and references are given in Table S2.

F_9_ progeny from 201 Da(1)-12×Ei-2 RILs was genotyped with 84 markers. Out of a total of 16,884 PCRs, only 126 failed or yielded ambiguous results. Residual heterozygosity was low, although the observed value of ca. 1.2% was larger than 0.4% which is expected for F_9_ progeny obtained by repeated selfing, possibly indicating some heterozygote advantage. Also, a significant deviation from expected 1:1 genotype frequencies was observed for a large segment of chromosome 1, comprising markers *C1P12* to *B12* (**Figure 2**), with the most significant excess of the Da(1)-12 genotype at marker *F1K23_2* (χ2 = 37.05, df = 1, P<0.001). Such distortion has also been observed for other RIL populations [2], [3], and may have been caused by unintentional selection during RIL generation. Nonetheless, the order of all genetic markers in the Da(1)-12×Ei-2 RILs was compatible with their physical position in the Col-0 sequence.

### QTL for Resistance against Crucifer Specialist Insect Herbivores

**Figure 3:**
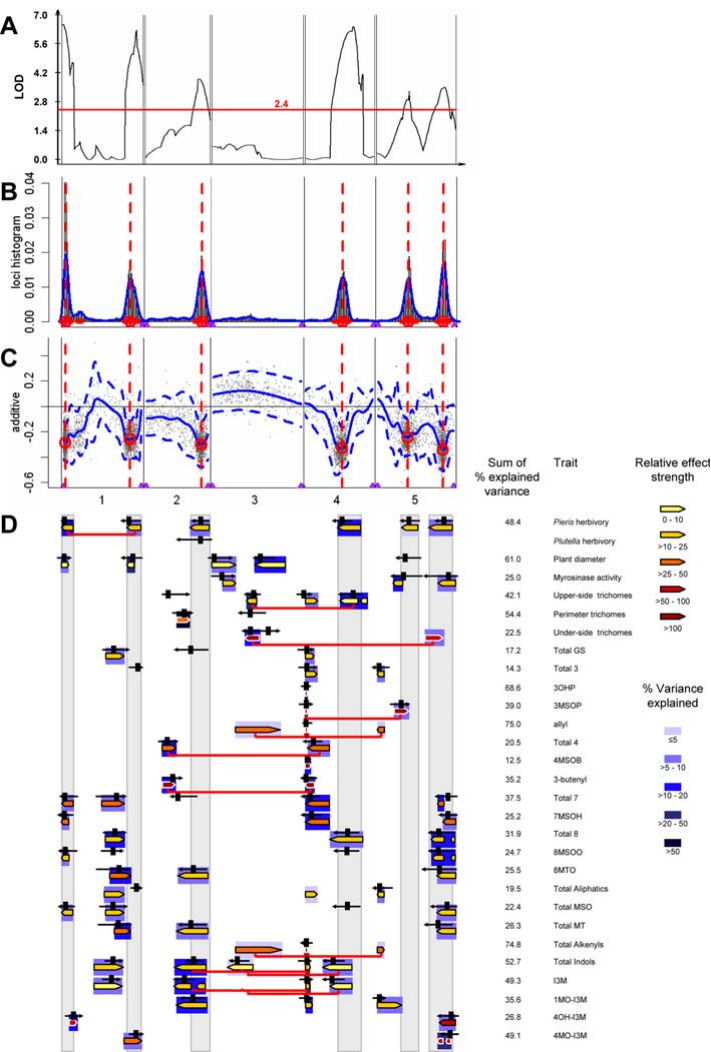
QTL for insect resistance and defense-related traits in Da(1)-12×Ei-2 recombinant inbred lines. A. QTL for resistance against *Pieris brassicae*, obtained with composite interval mapping (CIM). The horizontal red line indicates the significance threshold for this trait. B. Bayesian interval mapping (BIM) detects the same QTL as CIM. Horizontal red lines indicate high density probability regions in BIM, vertical red lines high density probability peaks. C. Additive effects in BIM, shown as a scatter plot with a smoothing spline fit (solid blue line) plus or minus two standard errors (dashed blue lines). For all six QTL, *Pieris* larvae cause greater damage when plants carry the Ei-2 alleles at the QTL. Hence, the Da(1)-12 alleles confer higher resistance. D. QTL for resistance against specialist lepidopterans, plant diameter, myrosinase activity, trichome density on the leaf upper and under-sides and perimeter, and for glucosinolates. For glucosinolates, QTL for individual compounds and for sum variables are given. Abbreviations are as follows: 3OHP = 3-hydroxypropyl; 3MSOP = 3-methylsulfinylpropyl; 4MSOB = 4-methylsulfinylbutyl; 7MSOH = 7-methylsulfinylheptyl; 8MSOO = 8-methylsulfinyloctyl; 8MTO = 8-methylthiooctyl; I3M = indol-3-yl-methyl; 1MO-I3M = 1-methoxy-indol-3-yl-methyl; 4OH-I3M = 4-hydroxy-indol-3-yl-methyl; 4MO-I3M = 4-methoxy-indol-3-yl-methyl. Total 3, 4, 7 and 8 are the sums of homomethionine-, di-homomethionine-, penta-homomethionine-, and hexa-homomethionine-derived glucosinolates, respectively. Total MSO, MT, and alkenyls are the sums of aliphatic glucosinolates with methylsulfinyl-, methylthio-, and alkenyl-groups, respectively. Total aliphatics and indoles are the sums of methionine- and tryptophan-derived glucosinolates, respectively, and total GS is the sum of all glucosinolates. Colored arrows correspond to 2-LOD support intervals for QTL identified with CIM, black arrows for high density probability regions in BIM, with vertical black bars showing the position of the high density probability peaks. Arrow directions correspond to effect directions; arrows pointing left indicate that the Ei-2 allele has a stronger effect on a particular trait. Arrow fill colors code for the relative effect strength of a QTL, arrow background colors for *R* ^2^, the percentage of variance explained by a QTL. Horizontal red lines connecting colored arrows indicate epistatic interactions between QTL.

We identified six QTL for resistance against *P. brassicae* herbivory, each two on chromosomes 1 and 5, and each one on chromosomes 2 and 4 (**Figure 3**). At all QTL, the Da(1)-12 genotype confers higher resistance to *P. brassicae*, and each Da(1)-12 allele increases resistance by 10–20%, consistent with higher resistance in the Da(1)-12 parental line (**Figure 1**). Together, these QTL explain nearly half of the phenotypic variance in our experiments (*R* ^2^ = 48.4%). In contrast, we did not find any QTL for resistance against *P. xylostella* herbivory with composite interval mapping (CIM), while Bayesian interval mapping (BIM) indicated the presence of one weak QTL, located at the same position as the *Pieris* herbivory QTL on chromosome 2 (**Figure 3**).

We tested for epistasis between major herbivory QTL but detected a significant interaction only between markers *C1P12* and *MSAT1.1* (*F* = 6.01, *df* = 1, *N* = 180, *P*<0.05) which correspond to the two QTL on chromosome 1. Here, a Da(1)-12 allele at *MSAT1.1* reduces plant damage by ca. 18% when plants carry the Da(1)-12 allele at *C1P12*, but only by ca. 4% when the allele at *C1P12* is Ei-2. *Vice versa*, a Da(1)-12 allele at *C1P12* reduces damage by ca. 20% in plants with a Da(1)-12 allele at *MSAT1.1* but only by ca. 5% in plants with an Ei-2 allele at *MSAT1.1*.

### Confirmation of a *Pieris* Resistance QTL with a Heterogeneous Inbred Family Strategy

We used a heterogeneous inbred family (HIF) strategy [28] to confirm the *Pieris* resistance QTL near marker *C1P12*. This strategy utilizes residual heterozygosity in a RIL population, and compares the phenotypes of genotyped progeny from a line heterozygous at a QTL candidate marker. HIF allows the rapid generation of a plant family homozygous for the majority of the genome but segregating at the candidate QTL.

We chose RIL DE196 which was heterozygous at both *Pieris* QTL on chromosome 1 (**Table S1**). From progeny of this line, we selected plants that carried either only Da(1)-12 or only Ei-2 alleles at marker *MSAT1.1*, but segregated at *C1P12*. Our statistical model accounted for plant size, flat and position effects. As expected, plants with a Da(1)-12 allele at *C1P12* experienced significantly less damage in *P. brassicae* herbivory screens than plants with an Ei-2 allele at this locus when *MSAT1.1* was homozygous Da(1)-12 (*F* = 18.70; *df* = 1; *N* = 130, *P*<0.05) but not when *MSAT1.1* was homozygous Ei-2 (*F* = 0.37; *df* = 2, *N* = 176, n.s.).

### QTL for Trichome Density

Leaf hairs, or trichomes, can contribute to plant defense against herbivorous insects in Arabidopsis [29], [30] and related plant species [31]. Therefore, we mapped QTL for trichome numbers on the leaf upper and under-side surfaces and the leaf perimeter (**Figure 3**). We identified three QTL controlling trichome density on the leaf upper sides, one on chromosome 3 and two with opposing effect on chromosome 4. These QTL may correspond to trichome QTL that have been mapped in several other Arabidopsis recombinant inbred lines [32]. For trichomes on the leaf perimeter, we identified only one QTL on chromosome 2. This QTL maps to approximately the same position as a major trichome QTL previously identified in the Col×L*er* RIL population [33]. Two QTL with opposing effects control trichome density on the leaf under-side, located on chromosomes 3 and 5, with the one in the center of chromosome 3 sharing its position with a QTL for trichome density on the leaf upper-side. Finally, we found epistatic interactions for both upper- and under-side trichomes. For trichome numbers on the leaf upper-side, we found a significant interaction (*F* = 4.13, *df* = 1, *N* = 94, *P*<0.05) between markers *3FM12*, close to the QTL LOD peak on chromosome 3, and *MSAT4.18*, which corresponds to the QTL near the center of chromosome 4. Trichome numbers were highest when both markers carried Ei-2 alleles and lowest when both markers had the Da(1)-12 genotype. A Da(1)-12 allele at *MSAT4.18* reduced trichome numbers on the leaf upper-side by ca. 17% when the allele at *3FM12* was Da(1)-12 but only by ca. 3.5% when *3FM12* had the Ei-2 allele. Likewise, the two QTL for leaf under-side trichomes interacted epistatically (*F* = 5.49, *df* = 1, *N* = 92, *P*<0.05). Here, trichome numbers were highest when *3FM12* carried the Ei-2 allele and *MIO24*, on chromosome 5, had the Da(1)-12 allele. Substitution of the Da(1)-12 allele at *MIO24* with an Ei-2 allele resulted in a reduction of trichome numbers by ca. 64%, substitution of the Ei-2 allele at *3FM12* in a reduction by ca. 87%. Finally, substitution of both alleles with the reciprocal genotypes led to a reduction by ca. 96% such that trichomes were rarely detected on the leaf under-side of RILs with a Da(1)-12 allele at *3FM12* and an Ei-2 allele at *MIO24*.

### QTL for Myrosinase Activity

We have identified three QTL for myrosinase activity that exceeded the significance threshold, one on chromosome 3, and two on chromosome 5 (**Figure 3**). At the QTL near the top of chromosome 3, the Ei-2 genotype confers higher myrosinase activity, while at the other QTL the Da(1)-12 alleles are more active. In a previous study, Mitchell-Olds and Pedersen [34] had identified two different myrosinase QTL in the Col×L*er* RIL population, one on chromosome 1 and the other near the center of chromosome 3. These QTL map to different locations than the ones identified in the present work. However, two of the known myrosinase genes in *Arabidopsis thaliana, TGG1* and *TGG2* [35], [36], map close to the LOD peak of the first myrosinase QTL on chromosome 5 (**Figures 2** **,** **3**), and likely cause this QTL. The third known myrosinase gene, *TGG3*, is a pseudogene in all accessions investigated so far [37], and does not map within the 2-LOD support interval of the second QTL on chromosome 5, although it is located in its vicinity. Hence, two of the QTL identified in Da(1)-12×Ei-2 represent novel myrosinase QTL.

### QTL for Aliphatic Glucosinolates

Ei-2 leaves produce ca. 50 – 60% more total glucosinolates than Da(1)-12 leaves, but this difference is small compared to the variation present among *A. thaliana* accessions (**Figure 1**; [18]). Only few QTL control total glucosinolate quantity, one on chromosome 1 and one near the top of chromosome 4 (**Figure 3**), which very likely corresponds to *AOP* (**Figure 1**; [14]). Total aliphatic glucosinolate accumulation is influenced by three QTL; *AOP*, a QTL near the bottom of chromosome 1, and a QTL near the top of chromosome 5. While a QTL for aliphatic glucosinolates near the top of chromosome 5 has also been identified in the L*er*×Cvi [12] and Col×L*er* RILs [6], the QTL near the bottom of chromosome 1 was previously unknown. The *AOP* locus (or a closely linked gene) also constitutes a QTL for nearly all individual aliphatic glucosinolates except for the hexa-homomethionine-derived glucosinolates, 8-methylsulfinyloctyl and 8-methylthiooctyl glucosinolate.

**Figure 4:**
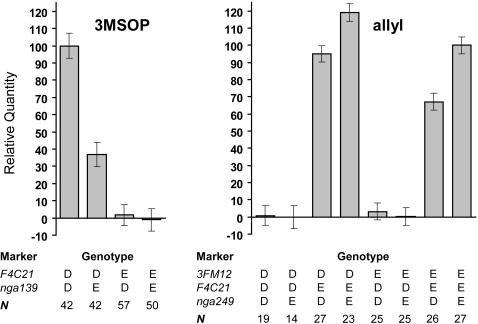
Epistatic interactions in the biosynthesis of homomethionine-derived glucosinolates. Left: 3-methylsulfinylpropyl glucosinolate (3MSOP) is produced when the genotype at the *AOP* locus (marker *F4C21*) is Da(1)-12. An Ei-2 allele at marker *nga139* reduces the accumulation of this glucosinolate by 60%. Right: allyl glucosinolate is produced when the genotype at *AOP* is Ei-2. A Da(1)-12 allele at *3FM12* increases and a Da(1)-12 allele at *nga249* decreases leaf allyl glucosinolate accumulation. *N* indicates the number of RILs with a particular combination of genotypes at the different markers.

Because both parental lines lack a functional *MAM1* gene, most aliphatic glucosinolates are homomethionine-derived, with only small quantities of di-homomethionine-derived glucosinolates being detectable. However, the genetic architecture underlying the biosynthesis of short chain aliphatic glucosinolates (*i.e.*, homo- and di-homomethionine derivatives) is nonetheless complex. The side chain structure of homomethionine-derived glucosinolates is modified by alleles at the *AOP* locus. RILs with the Da(1)-12 *OHP* allele accumulate 3-hydroxypropyl and 3-methylsulfinylpropyl glucosinolates, while lines with the Ei-2 *ALK* allele accumulate allyl glucosinolates. Therefore, QTL effects for 3-hydroxypropyl and 3-methylsulfinyl glucosinolates have the same direction, but are opposite to the QTL effect for allyl glucosinolate (**Figure 3**). The quantity of 3-methylsulfinylpropyl glucosinolate is also influenced by a QTL on chromosome 5, and by an epistatic interaction between this QTL and *AOP*. The QTL on chromosome 5 is located near marker *nga139*, in the vicinity of the *MAM* genes (**Figure 2**). Among the RILs that harbor the Da(1)-12 allele at *AOP* and are, thus, capable of producing 3-methylsulfinylpropyl glucosinolate, those lines with a Da(1)-12 allele at *nga139* accumulate two- to threefold more 3-methylsulfinylpropyl glucosinolate than lines with an Ei-2 allele at this marker (**Figure 4**).

Likewise, allyl glucosinolate accumulation is not only determined by *AOP* but also by two further QTL, on chromosome 3 (marker *3FM12*) and near the top of chromosome 5 (marker *nga249*), and by pairwise epistatic interactions between *AOP* and these QTL (**Figure 4**). Allyl glucosinolates are only synthesized when RILs harbor the Ei-2 allele at *AOP*, with a Da(1)-12 allele at marker *3FM12* increasing and a Da(1)-12 allele at marker *nga249* decreasing allyl glucosinolate accumulation. Since allyl glucosinolates account for the majority of alkenyl glucosinolates in Da(1)-12×Ei-2 leaves, total alkenyl glucosinolates follow the same pattern of QTL and epistatic interactions.

Within the *MAM* gene cluster, *MAM1* and/or *MAM2* control variation in short-chain aliphatic glucosinolate accumulation [7], [27]. The third gene in this cluster, *MAM-L*, is essential for the production of long chain glucosinolates, and a *MAM-L* knock-out abolished the formation of long chain aliphatic glucosinolates [38]. With the possible exception of 3-methylsulfinylpropyl glucosinolate, the *MAM* locus has no detectable influence on glucosinolate profile variation in Da(1)-12×Ei-2 (**Figure 3**). Nonetheless, in other regions of the genome we found several QTL for long chain aliphatic glucosinolates, *i.e.* for penta- and hexa-homomethionine-derived glucosinolates. Two QTL are located on chromosome 1, two on chromosome 4, and one is positioned near the bottom of chromosome 5. Hence, these additional QTL control variation in the accumulation of long-chain aliphatic glucosinolates in Da(1)-12×Ei-2, even though the *MAM* locus does not contribute to this variability.

### QTL for Indole Glucosinolates

We identified five QTL for total indole glucosinolate accumulation, each one on chromosomes 1, 2 and 3, and two on chromosome 4. The QTL near the top of chromosome 4 had the strongest effect on indole glucosinolate accumulation. This QTL co-localizes with the *AOP* locus, suggesting that *AOP* influences not only aliphatic glucosinolates but also indole glucosinolate accumulation, either directly by catalyzing a biosynthetic reaction step or indirectly via utilization of a pool of metabolites that is shared in aliphatic and indole glucosinolate biosynthesis. Alternately, a gene tightly linked to *AOP* could also explain this indole glucosinolate QTL.

We also detected complex epistatic interactions between the QTL for total indole glucosinolate accumulation. We found pairwise epistatic interactions between the QTL on chromosome 3 (marker *MSAT3.19*) and the QTL near the bottom of chromosome 2 (marker *C22*), between *MSAT3.19* and the QTL near the top on chromosome 4 (marker *F4C21*), and between *MSAT3.19* and the QTL at the center of chromosome 4 (marker *T6G15*), as well as a triple interaction between *C22*, *MSAT3.19* and *F4C21* (**Figure 3**, **Table 1**).

**Table 1:** Epistatic interactions in glucosinolate biosynthesis

Trait	Source	*d.f.*	*F*-ratio	*P*
3-methylsulfinylpropyl	*F4C21*	1	102.54	0.00000
	*nga139*	1	23.81	0.00000
	*F4C21*×*nga139*	1	20.17	0.00001
	Error	187		
allyl	*3FM12*	1	8.59	0.00382
	*F4C21*	1	632.11	0.00000
	*nga249*	1	12.54	0.00051
	*3FM12*×*F4C21*	1	10.99	0.00111
	*3FM12*×*nga249*	1	0.20	0.65704
	*F4C21*×*nga249*	1	16.54	0.00007
	*3FM12*×*F4C21*×*nga249*	1	0.51	0.47812
	Error	178		
Total 4	*F3K23*	1	23.02	0.00000
	*nga8*	1	20.58	0.00001
	*F3K23*×*nga8*	1	9.88	0.00195
	Error	182		
3-butenyl	*F3K23*	1	20.77	0.00001
	*F4C21*	1	56.73	0.00000
	*F3K23*×*F4C21*	1	15.06	0.00015
	Error	181		
Total Indoles	*B12*	1	11.49	0.00090
	*C22*	1	17.21	0.00006
	*MSAT3.19*	1	14.35	0.00022
	*F4C21*	1	31.28	0.00000
	*T6G15*	1	6.02	0.01534
	*C22*×*B12*	1	1.68	0.19748
	*MSAT3.19*×*B12*	1	0.30	0.58454
	*F4C21*×*B12*	1	0.40	0.52740
	*T6G15*×*B12*	1	0.00	0.96366
	*MSAT3.19*×*C22*	1	8.33	0.00450
	*F4C21*×*C22*	1	0.04	0.84950
	*T6G15*×*C22*	1	0.01	0.94323
	*F4C21*×*MSAT3.19*	1	6.36	0.01279
	*T6G15*×*MSAT3.19*	1	7.27	0.00783
	*T6G15*×*F4C21*	1	0.55	0.46085
	*MSAT3.19*×*C22*×*B12*	1	0.00	0.96727
	*F4C21*×*C22*×*B12*	1	1.01	0.31703
	*T6G15*×*C22*×*B12*	1	0.00	0.96243
	*F4C21*×*MSAT3.19*×*B12*	1	0.17	0.68519
	*T6G15*×*MSAT3.19*×*B12*	1	0.73	0.39366
	*T6G15*×*F4C21*×*B12*	1	0.41	0.52310
	*F4C21*×*MSAT3.19*×*C22*	1	13.69	0.00031
	*T6G15*×*MSAT3.19*×*C22*	1	2.05	0.15448
	*T6G15*×*F4C21*×*C22*	1	0.23	0.63376
	*T6G15*×*F4C21*×*MSAT3.19*	1	0.69	0.40783
	Error	144		

The QTL pattern for indol-3-yl-methyl glucosinolate, the most abundant indole glucosinolate in *A. thaliana* leaves [18], reflects largely the QTL pattern for total indole glucosinolate accumulation, except that the QTL for total indole glucosinolates on chromosome 3 did not exceed the significance threshold for indol-3-yl-methyl glucosinolate. Nonetheless, marker *MSAT3.19* showed the same pattern of epistatic interactions for indol-3-yl-methyl glucosinolates as for total indole glucosinolates, when we included it in our statistics models.

Da(1)-12×Ei-2 leaves synthesize three further indole glucosinolates, 1-methoxy-indol-3-yl-methyl, 4-hydroxy-indol-3-yl-methyl and 4-methoxy-indol-3-yl-methyl glucosinolate. 1-methoxy-indol-3-methyl glucosinolate shares two of its three QTL with indol-3-yl-methyl glucosinolate but has an additional QTL near the top of chromosome 5. This additional QTL might correspond to the position of a gene responsible for the generation of the methoxy-group at position 1 of the tryptophan moiety.

4-hydroxy-indol-3-yl-methyl glucosinolate accumulation is controlled by two QTL, one near the top of chromosome 1 and the second near the bottom of chromosome 5 (**Figure 3**). The position of this QTL on chromosome 5 and its effect direction are shared by one of the QTL controlling 4-methoxy-indol-3-yl-methyl glucosinolate accumulation, suggesting a biosynthetic connection between 4-hydroxy-indol-3-yl-methyl and 4-methoxy-indol-3-yl-methyl glucosinolate. However, the second QTL for 4-methoxy-indol-3-yl-methyl glucosinolate is located at a different position, near the bottom of chromosome 1, while the second 4-hydroxy-indol-3-yl-methyl glucosinolate QTL maps near the top of chromosome 1.

## Discussion

### Is Herbivory by *Pieris brassicae* Influenced by Variation in Glucosinolates, Myrosinase Activity or Trichomes?

QTL for different traits may be considered to co-localize when their 2-LOD support intervals overlap. Co-localization of QTL does, of course, not prove that these QTL are caused by the same gene. Likewise, linked QTL for different traits may have the same cause, even when their 2-LOD support intervals do not overlap, due to the complexity of the statistics that guide QTL mapping. Nonetheless, co-localization of QTL for different traits may provide an approximation for comparing the genetic architecture underlying different but potentially related traits.

We have identified six QTL for resistance against *P. brassicae*, each two on chromosomes 1 and 5, and each one on chromosomes 2 and 4. For many of the traits that we investigated because they are known to influence resistance against generalist insect herbivores, we found one or more QTL that co-localize with *Pieris* resistance QTL. However, one of the three myrosinase activity QTL does not co-localize with a *Pieris* resistance QTL. Only one of the three QTL for leaf upper-side trichomes, on chromosome 4, co-localizes with a resistance QTL but this QTL shows the wrong sign for its effect. One would expect that trichomes provide physical resistance against insect herbivory and, thereby, reduce plant damage. However, plant damage increases with increasing numbers of leaf trichomes controlled by this locus. None of the leaf perimeter trichome QTL co-localizes with a resistance QTL, and for QTL controlling leaf under-side trichomes, only one of two QTL co-localizes.

For total glucosinolate accumulation, we identified two QTL, but neither co-localizes with a *Pieris* resistance QTL. Likewise, none of the QTL for total aliphatic glucosinolate accumulation co-localizes with a resistance QTL, and only two of the five QTL for total indole glucosinolate accumulation co-localize with herbivory QTL. A similar picture emerges for QTL controlling sums of homomethionine, di-homomethionine, penta-homomethionine and hexa-homomethionine-derived glucosinolates or for QTL controlling total methylthio, methylsulfinyl or alkenyl glucosinolates (**Figure 3**). Hence, sum variables for glucosinolate classes do not account for resistance against *P. brassicae*. But also QTL for individual glucosinolates do not explain the QTL for *Pieris* resistance. The QTL with the largest impact on glucosinolate profiles, *AOP*, does not co-localize with any of the resistance QTL. And for all individual glucosinolates, either one or more QTL do not co-localize with herbivory QTL, or at least one QTL has a different sign for its effect than the others, while all *Pieris* QTL have the same sign for their effect, with the Da(1)-12 allele improving resistance against herbivory by *Pieris* larvae. Finally, none of the *Pieris* QTL maps near the two major loci that specify glucosinolate hydrolysis product identity, *ESP* [5] and *ESM1* [8] (**Figure 2**), although both parental lines, Da(1)-12 and Ei-2, display sequence polymorphisms in the *ESP* genomic region that correlate with *ESP* expression [5]. Hence, we conclude that none of the investigated traits, trichomes, myrosinase activity or glucosinolate accumulation, appears to cause the QTL for *Pieris* resistance in Da(1)-12×Ei-2 RILs. Further fine-mapping and, ultimately, cloning of the underlying genes causing the *Pieris* resistance QTL is necessary to help understand how Arabidopsis and other crucifers defend themselves against specialist insect herbivores such as *P. brassicae*.

### Prospects of Improving Insect Resistance in Cruciferous Crops

*Pieris brassicae* and other Pieridae are some of the most serious pests on cruciferous crop plants such as rapeseed, cauliflower, or broccoli [39]–[41]. We have analyzed herbivory by *P. brassicae* larvae with a new RIL population, obtained from a cross between the parental lines Da(1)-12 and Ei-2. We found no detectable effect of glucosinolates or myrosinase activity on larval herbivory, indicating that the variation in the glucosinolate-myrosinase system that is present in our RIL population does not contribute to variation in plant damage caused by *Pieris* larvae. Nonetheless, the glucosinolate-myrosinase system does play a role in the interaction between *P. brassicae* and *A. thaliana* or other Brassicaceae: Adult *Pieris* females use glucosinolates and their hydrolysis products to locate host plants for oviposition, and hydrolysis products have a stimulating effect on oviposition for *P. brassicae* and other Pieridae [24], [42]–[45]. Likewise, glucosinolate breakdown products serve as a stimulant for larval feeding initiation [46]–[48]. This may explain why herbivory by *Pieris rapae*, a close relative of *P. brassicae*, is significantly reduced in *tgg1 tgg2* double mutants which have very low levels of Arabidopsis wild type myrosinase activity [22]. Hence, a reduction of glucosinolate levels or myrosinase activity in cruciferous crops could potentially reduce plant damage caused by *P. brassicae*. However, a decrease in the effectiveness of the glucosinolate-myrosinase system would very likely render crucifer crops more susceptible to generalist insect herbivores which are sensitive towards glucosinolate-based defenses [5]–[9], [22]. Furthermore, such a manipulation of the glucosinolate-myrosinase system bears the risk that plants could become more attractive to herbivores which usually do not consume crucifers because these insect species have no effective means to withstand toxic products originating from glucosinolate hydrolysis. Thus, manipulating the glucosinolate-myrosinase system to increase resistance against insect herbivores may be problematic. The detection of QTL that appear to be independent of the glucosinolate-myrosinase system may provide a way to solve this dilemma. Manipulating the genes that underlie the detected resistance QTL could help increase crop protection against *P. brassicae*, without interfering with a complex defense system that protects crucifers effectively against most herbivorous insects.

## Materials and Methods

### Plant and Insect Growth Conditions

*Arabidopsis thaliana* plants for RIL development were grown under continuous light, supplied by Osram Fluora L36/W77 neon bulbs with an intensity of 150 µmol s^−1^ m^−2^ at 20°C and 60% relative humidity in an environment-controlled growth room. Plants for insect herbivory trials, leaf glucosinolate extraction, and myrosinase assays were grown in 11.5 h day/12.5 h night cycles at 22°C and 60% relative humidity (day conditions), and 16°C and 80% relative humidity (night conditions) in an environment-controlled growth room. Here, light was supplied by NH 360 FLX Sunlux ACE bulbs with an intensity of 200 µmol s^−1^ m^−2^. Plants were grown in an autoclaved 1:3 vermiculate/potting soil mix with 20 ml time-release fertilizer (Osmocote) per flat. After sowing into damp potting medium, flats were covered with clear plastic grow domes, and seeds were stratified for 3–4 days at 6°C in the dark. In general, seeds germinated within 2–3 days, and grow domes were removed 5 days after transfer to the light. Then seedlings were transferred to fresh soil in 96-celled 32.5×51 cm2 flats at a density of 1 seedling per cell. All assays were carried out with 3-week old plants.

*Pieris brassicae* eggs were obtained from Seritech (Warwick, UK). After hatching, insects were reared on *Brassica napus var. oleifera* for 2–3 days before the experiments. *Plutella xylostella* eggs were obtained from New York State Agricultural Experiment Station Geneva, NY, USA, and a colony was maintained at the Max Planck Institute for Chemical Ecology, Jena, Germany. Insects were raised on artificial diet according to published procedures [49].

### Generation of Da(1)-12×Ei-2 Recombinant Inbred Lines

Da(1)-12 (accession no. N917) and Ei-2 (N1124) accessions were obtained from the Arabidopsis stock center (Nottingham, U.K.). Except for the initial cross between both accessions, all following generations were propagated by selfing. 215 F_2_ plants were randomly selected from the progeny of a single heterozygous F_1_ plant. For every advanced generation, 4–8 seeds per line were planted, and a single plant was randomly chosen from each line for seed production. Finally, seeds from a single F_8_ plant per line were bulk-collected resulting in a final set of 201 Da(1)-12×Ei-2 RILs. This new RIL population will be made available through the Arabidopsis stock centers.

### DNA Extraction, Genotyping, Genetic Map

DNA was isolated as described in [17]. Molecular markers were obtained from publicly available sources (http://www.arabidopsis.org; http://www.inra.fr/qtlat, [49]), or were generated from microsatellite loci identified in the Col-0 genome sequence [51]. More than 150 potential markers were tested for polymorphism between parental lines, using DNA from Da(1)-12, Ei-2, and a 1∶1 mixture of DNAs from both lines. 84 PCR products were found suitable for genotyping (**Table 2**), and allowed to distinguish between Da(1)-12 and Ei-2 genotypes, and between homozygous and heterozygous loci on 4% Metaphor agarose (Biozym diagnostics, Germany). PCR reactions contained, in general, ca. 30 ng DNA, 2.3 µl 10x PCR buffer (Qiagen, Germany), 4 nmol of each dNTP, 1.25 pmol of each of both primers, 70 nmol MgCl_2_, and 0.15 U *Taq* DNA polymerase (Qiagen, Germany) in a 23 µl volume. Cycling conditions were 94°C for 2 min, followed by 38 cycles of 94°C for 15 s, 50°C or 55°C for 15 s, and 72°C for 30 s, with a final extension of 72°C for 2 min on an Applied Biosystems 9700 Thermocycler. Genotyping was carried out with DNA extracted from individual F_9_ progeny. A genetic map was constructed with MAPMAKER/EXP Version 3.0 [52]. Genotype data for the Da(1)-12×Ei-2 RILs are available in **Table S1**.

**Table 2:** Markers used for genotyping of Da(1)-12×Ei-2 RILs

Marker	Chr	BAC/P1	Primer 1 (5′→3′)	Primer 2 (5′→3′)	Gel pattern
***C1P12***	1	F20B24	CTGGAAGTCCATACCATGAG	GTTCGTCGTTCGTGGTATTG	D>E
***F20D23_2***	1	F20D23	CCGTCACACCATTCACAATC	CCAACCCCTTATATATCGTTC	D>E
***MSAT1.7*** a	1	F12K8	GCTTTTATCAGCTCAAACAT	ACTCTTACGTTTGGAGTTCA	D>E
***NGA248*** a	1	F3H9	TCTGTATCTCGGTGAATTCTCC	TACCGAACCAAAACACAAAGG	D<E
***F1K23***	1	F1K23	GAACCAATAAGGAGGCTCAAC	CCATACGGAGAAACCTTCTTC	D>E
*F1K23_2*	1	F1K23	CAATTTCGAGTTTCGGATTTTC	CTTCACATCAATGCTTGTAATAG	D<E
***MSAT1.4*** a	1	F28L22	CTAAACTAGAACCAGGGGTAA	ACAAAAATCGTGGTGATAATA	D<E
***T27K12*** a	1	F7F22	GGAGGCTATACGAATCTTGACA	GGACAACGTCTCAAACGGTT	D>E
***B14***	1	F11F12	CCATTCTCGTCGTGTTATAAG	GAAATGTTAAGGCCAAAATACAG	D<E
***B12***	1	T18A20	CAACTCGTTATAACAGGTTTTAC	CCAAATACTAAAGAGGGAATTG	D<E
***F5I14*** a	1	F5I14	CTGCCTGAAATTGTCGAAAC	GGCATCACAGTTCTGATTCC	D>E
***MSAT1.12*** a	1	T26J14	TTAGAGATTCGCCAACCTC	CGTGTGCCCAACCA	D>E
***MSAT1.13*** a	1	F24J5	GTCAAACCAGTTCAATCA	CAACCACCAGGCTC	D<E
***MSAT1.1*** a	1	F20P5	ATACGATAAGATTTATTAGCA	CCCATGCTCTTTTTGTGAAA	D>E
***MSAT1.2*** a	1	F22K20	TTGAGTGGTGCCGCTTG	ATATCTCCATCGCTGCAACC	D>E
*MSAT2.38* a	2	F18P14	TGTAACGCTAATTTAATTGG	CGCTCTTTCGCTCTG	D>E
***MSAT2.18*** a	2	T4E14	TAGTCTCTTTTGGTGCGCATA	AGCCTCTCCAAGCTTAGGTCT	D>E
***MSAT2.28*** a	2	T26I20	AATAGAAATGGAGTTCGACG	TGAACTTGTTGTGAGCTTTG	D<E
***MSAT2.11*** a	2	F19F24	GATTTAAAAGTCCGACCTA	CCAAAGAGTTGTGCAA	D>E
***T20K24***	2	T20K24	CAATATTCGTGGGAGTTAGTC	GCTGTCGAATTACATTTCTTTAC	D<E
***F3K23***	2	F3K23	CTCGCAGCGTCTGCAAATTC	GAAGCGGAAGATGGAGAGAC	D>E
*MSAT2.31* a	2	T22F11	GCTCCTCTTTGCCGCTAG	GCGATTTCATCTTGTGCATC	D>E
***MSAT2.37*** a	2	T19L18	GGTTGTTTCATCGAAAGCA	CATGGTCTCGCTGGTGTAT	D<E
***C22***	2	T26B15	CTTGGCAACTTCATTCAATTTC	GAAAGTAGAGAAGCATTTAGAC	D<E
*MSAT2.4* a	2	T26B15	TGGGTTTTTGTGGGTC	GTATTATTGTGCTGCCTTTT	D<E
***A21***	2	T1J8	CCATCTAAACTGCTTACGATG	GTGACCCATTCTTCTCTTTTC	D<E
***MSAT2.22*** a	2	F17A22	CGATCCAATCGGTCTCTCT	TGGTAACATCCCGAACTTC	D<E
***C3P41***	3	F9F8	GGTCGTATCCTCTTATCGAAC	CTTGTGAGTGGTCTTATGAAAG	D<E
***3FM08***	3	K20I9	GGTTCGTATCCAAAAACCAAG	CCATCATTGGAGCAAGAGAC	D>E
***C3P43***	3	MRC8	CAATGTTGGCTTGGAAATAATG	CATTGCCGGTAAAAATGTTTTTC	D>E
***3FM09***	3	MAL21	CTAATTACTATGGCGGAGAATTC	CTAAAGAAATCTGCGGTCTTC	D>E
***3FM10***	3	MSD21	CATTACTTCACTGTTGCTTTAC	GACAGGTTATGGCTTGTTAATC	D>E
***MSAT3.19*** a	3	K7M2	TAATTCGATCCAATTGACAT	TGGCTTGGCACAAAC	D<E
***3FM12***	3	K24A2	TAGGGAAGCATTTGTCTTGAG	TGCTTAAAGTGACGGTAAAATG	D: 1 band E: 2 bands
***MSAT3.8*** a	3	K5K13	ATGTTAAAAACCCGTGTTGG	TTTAACCTTATCCGGGAAAG	D>E
***MSAT3.32*** a	3	MXO21	GCACTTGCAGCTTAACTT	CGTGACTGTCAAACCG	D>E
***C32***	3	T15B3	GAAGAGGATGAACAAAGATAAG	CAAATCTGCCTCCTCCATAAG	D<E
***MSAT3.28*** a	3	T26I12	TACAAGTCATAATAGAGGC	GGGTTTAGCATTTAGC	D<E
*T5J8_2* b	4	T5J8	CGATCATCGGTGTTCACCTT	GAAAATAAATCGTCATATGGTGTACTG	D>E
*T5J8_1* b	4	T5J8	GCCAAGACGCAGAAGAAGAG	TCTCATTATTCCCCACAATGC	D<E
***F4C21***	4	F4C21	GCGCTTCATCTAGTTACGCTTT	CCCGGACTGAACCAAACTAA	D>E
***NGA8*** a	4	T32A17	GAGGGCAAATCTTTATTTCGG	TGGCTTTCGTTTATAAACATCC	D<E
***F28M11***	4	F28M11	CACCATATTGGCCTCAAATTG	CAAAAACCCGTTCCACCAAAC	D<E
***MSAT4.25*** a	4	F25E4	GAATGGTTGTTGATAGTTGA	AAATTTCAGGAGGTGATAGA	D<E
*F25G13*	4	F25G13	GCCAGGTTCTTTTCATTTCTC	GGGCGTTTAATTTGCATTCTTC	D<E
***MSAT4.35*** a	4	F25G13	CCCATGTCTCCGATGA	GGCGTTTAATTTGCATTCT	D<E
***T6G15***	4	T6G15	GTAGCCAGAGATGGAAGTTAC	GGGTCCTTACTGAGGCTTTG	D>E
***MSAT4.18*** a	4	T12H17	TGTAAATATCGGCTTCTAAG	CTGAAACAAATCGCATTA	D<E
***T27E11***	4	T27E11	GTGATTCCCGTCTGCTAAAC	CCTCCTTCAGCATCATAGTG	D>E
***F25O24***	4	F25O24	CAATGTATTTGGATGTGTTTGTTC	GGATGGTAACACGGCTAAAC	D<E
***FM12***	4	T16L4	GAAGCCCCTATGAGATGGTC	GTGAGGGAGTTAGGTAGCAAC	D<E
***FM14***	4	F9N11	TCAAGGGAGACTTGGAGAAC	GGGATCGTTATGCACTTGTTTG	D<E
***FM17***	4	F9N11	CTCCCTCTTCGGAGAAATTC	CATCTCTTATAGGCCTCTCTC	D>E
***4FM05***	4	F17I23	CTAACAGATTTGGTGAATAACAAG	TCATTTGATGTGCCAGTAAATC	D>E
***FM21***	4	T10C21	CTCAAGCGGTGGAAATTGGAG	GTAAAGAATGTCCAGGGCAG	D<E
***MSAT4.14*** a	4	F8F16	GACCGTTTCTAGTGCTCACA	ACGGAATAAGCGGAGGA	D<E
***FM24***	4	F3L17	GAGCATCCGCGTAGGTTAAG	CACAGAGAGACTCAAAAATACTG	D<E
*FM34*	4	F11C18	TGGTCTCTCAACTCCAACAC	CATTGAGATTTGAGCCAAACAG	D<E
*MSAT4.11* a	4	F10N7	AAAAATCCGGTAGAGCATCC	CCAATTCCGAGCCAGTAA	D<E
*F10N7*	4	F10N7	GTTGCTCGAAACCTCTCAATC	GCCTCACCGATACGTTTCTG	D<E
***4FM01***	4	F8B4	AGTAGATACAATGCGTTGACC	GGAGCGTTAATAGTGTGTATG	D<E
*FM29*	4	L23H3	GTCCAGGTTGCTGAAGAGAAG	GTATTGTTTGGTTGGTATGAGC	D<E
***MSAT4.9*** a	4	F4D11	GAAATCAACGGCTGAG	AAGTAATTAAGACGCTGAGA	D<E
***F4B14***	4	F4B14	CGTCGTTTATTTCACCACCAC	GGTACAAAGATGGGTTAAACTG	D<E
*F6G17*	4	F6G17	GACACGCAAACAAAGTAAAAGTC	GATGGTGACATAGACCCAATG	D>E
***MSAT4.33*** a	4	F6G17	TTCTTTGACACGCAAACA	TGGTGACATAGACCCAATG	D>E
***MSAT4.21*** a	4	F19F18	TTATGCTATGGCTGTTTGGT	CGAAATCTGTTCTTGCATTC	D>E
***MSAT4.30*** a	4	F20D10	AGAGCACTCACCGTTCAT	TGTGTTCGTGGATTTACC	D<E
***MSAT4.31*** a	4	T5J17	AGGGATATGGATTGAGA	GCCGTATAACTATTGGTT	D<E
***NGA249*** a	5	MAH20	TACCGTCAATTTCATCGCC	GGATCCCTAACTGTAAAATCCC	D>E
***NGA151*** a	5	F18022	GTTTTGGGAAGTTTTGCTGG	CAGTCTAAAAGCGAGAGTATGATG	D<E
***NGA139*** a	5	K18P6	GGTTTCGTTTCACTATCCAGG	AGAGCTACCAGATCCGATGG	D>E
***C5P71***	5	T26D3	GACGATGGTGGAGTGATAAG	CTTTGACCTCAAACTTAAGTAG	D<E
***MSAT5.25*** a	5	MOK9	GCTTAATTTGGGTTAAAT	GCACGCAAGTGACT	D<E
***MSAT5.22*** a	5	MWP19	AGAACAAGTTAGGTGGCT	GGGACAAGAATGGAGT	D<E
***C5P81***	5	MFO20	GTCAAAGAGTTACTCCGTTAC	CGAGACAAGAGCATGTTATATG	D<E
***MIO24***	5	MIO24	GTACAATAATTTAGAGAGTATTTTG	CTAGCTCAACTTACTGCTTAATG	D<E
***MNC6***	5	MNC6	GTTTGGGTCCAATGATAAAATC	GCCTATTGGGCTGAGTTTTC	D>E
***MMN10***	5	MMN10	CAGTGTCGGCTAATTTCGAC	CAGTCGACATTTCAAAGGTTC	D<E
***5FM10***	5	MFB13	GATTTGACGACTGATTACATAAC	GCTTGAAATTTGTGTGTATTGTC	D>E
***5FM09***	5	MPA24	CAATTTCTTGTTATCTGCTTATG	CCATTGCCATATGTTTCCCTC	D<E

a : Markers are from http://www.inra.fr/qtlat/msat/index.php

b : Markers are from [14]

Bold-typed markers were used for QTL mapping.

### Insect Herbivory Screens

*P. brassicae* herbivory screens were performed at 7 different times, and each experiment was carried out with at least 3 replicates per RIL. RIL replicates were completely randomized over 96-celled flats. At the beginning of each experiment, plant diameter was measured. One larva was placed on each plant rosette without prior starvation, and larvae were allowed to move freely. After 24 hours of herbivory, the leaf area removed by the insects was assessed visually, and an artificial scale was established to determine the percentage of removed rosette tissue. In total, more than 9000 data points were collected for *P. brassicae* herbivory. *P. xylostella* herbivory screens were performed similarly, except that larvae were starved for 6 hours prior transfer to plants, and larvae were allowed to feed for 2–3 days. Total sample size for *P. xylostella* herbivory exceeded 2400 plants.

### Trichomes

Trichome analysis was carried out with 96 RILs chosen to include lines with a maximum number of recombination breakpoints. Per RIL, 4 replicates were analyzed, and trichomes from the 3^rd^ to 6^th^ true leaves of 3-week old plants were counted with a Stemi SV6 binocular (Carl Zeiss, Jena, Germany). Every leaf was placed underneath the binocular such that the leaf tip touched the border of the visual field. This way, only the upper half of the leaf was visible and used for trichome analysis. Trichome numbers were counted for the leaf upper and under-sides and for the leaf perimeter.

### Glucosinolate Extraction and HPLC Analysis

Glucosinolates were extracted in a 96-well format as described in [18]. HPLC separation and identification of extracted desulfo-glucosinolates was carried out as described in [17]. In brief, HPLCs were run on a Hewlett Packard HP 1100 system (Agilent), equipped with a HP Lichrocart 250-4 RP18e 5 µm column. The elution was accomplished with a water (solvent A) – acetonitrile (solvent B) gradient using the following program: 1.5 – 5% (v/v) B (6 min), 5 – 7% B (2 min), 7 – 21% B (10 min), 21 – 29% B (5 min), 29 – 43% B (7 min), 43 – 92% B (0.5 min), 92% B (2.5 min), 92 – 1.5% B (0.5 min), 1.5% B (4.5 min). Desulfo-glucosinolates were identified according to retention time and UV spectra, and quantified from HPLC peak areas at A229 nm, using published response factors [53], [54] to correct for different UV absorption capacities of individual glucosinolates.

### Myrosinase Extraction and Analysis

Myrosinase extraction from 100 mg leaf tissue and UV-spectrophotometric activity assays were carried out as described in [22]. Two independent experiments were conducted, once with the complete RIL population, once with the 96 most informative RILs. Relative myrosinase activity was measured as a spectrophotometrical change at 227 nm through breakdown of sinigrin (allyl glucosinolate) within 15 min using a Multiskan Spectrum (Thermo Fisher Scientific, Germany) spectrophotometric plate reader.

### Quantitative Analyses

Systat Version 10 (SPSS Inc.) was used to analyze natural variation for insect resistance and related traits in the following 16 Arabidopsis accessions: Akita (Akita, Japan, Versailles identification no. 252 AV), Col-0, Ct-1 (Catania, Italy, N1094), Cvi-0 (Cape Verdi Islands, N902), Da(1)-12 (Czech Republic or Slowakia, N917), Edi-0 (Edinburgh, UK, N1122), Ei-2 (Eifel, Germany, N1124), Kn-0 (Kaunas, Lithuania, N1286), L*er*, Shahdara (Shahdara river (Pamir), Tadjikistan, N929), Mh-1 (Muehlen, Poland, N1368), Mt-0 (Martuba/Cyrenaika, Lybia, N1380), N13 (Konchezero, Russia, CS22491), Oy-0 (Oystese, Norway, N1436), Stw-0 (Stobowa/Orel, Russia, N1538), and Tsu-0 (Tsu, Japan, N1564). ANOVA was used to obtain least squares means for each accession for *P. brassicae* and *P. xylostella* herbivory, for plant diameter in *P. brassicae* herbivory experiments, for myrosinase activity, and for trichomes on the leaf upper and under-side surfaces and the leaf perimeter. For *P. brassicae* (N = 973) and *P. xylostella* herbivory (*N* = 912), the ANOVA model was TRAIT = CONSTANT+ACCESSION+EXPERIMENT+FLAT(EXPERIMENT)+COLUMN+ROW+PLANT DIAMETER. COLUMN and ROW are variables to control for position effects. These variables are particularly important in *P. brassicae* herbivory screens to compensate for larval movement during experiments (*F* = 19.18, *df* = 11, *N* = 973, *P*<0.000001 for COLUMN, and *F* = 41.67, *df* = 7, *P*<0.000001 for ROW). EXPERIMENT accounts for variation between experiment replicates, FLAT(EXPERIMENT) for variation between flats within an experiment. Similarly, least squares means were obtained for plant diameter in *P. brassicae* (*N* = 973) and *P. xylostella* (*N* = 912) herbivory screens with the model TRAIT = CONSTANT+ACCESSION+EXPERIMENT+FLAT(EXPERIMENT)+COLUMN+ROW. Myrosinase activity (*N* = 68), glucosinolates (*N* = 83) and leaf trichome density (*N* = 153) were evaluated in only one experiment each and all accessions were grown completely randomized within one flat. Therefore, the model was TRAIT = CONSTANT+ACCESSION+COLUMN+ROW to obtain least squares means for myrosinase activity. In this model, COLUMN and ROW are variables used to control for the time delay that occurs during the processing of individual microtiter plate positions in our plate reader. The model for the analysis of all individual glucosinolates was TRAIT = CONSTANT+ACCESSION. Glucosinolate sum variables were generated by summing up least squares means for individual glucosinolates after correcting for different UV absorption capacities with published response factors [52], [53]. Finally, least squares means for trichome density on the leaf upper and under-sides and the leaf perimeter were obtained with the model TRAIT = CONSTANT+ACCESSION+LEAF, with LEAF being a variable to control for a potential effect of leaf developmental stage on trichome density. For the Da(1)-12×Ei-2 RIL population, similar ANOVA models were used as described above, with a few exceptions: A variable FLAT was included in the glucosinolate and trichome models to account for variation between flats, and the variables EXPERIMENT and FLAT(EXPERIMENT) were included to control for variation between experimental replicates and between flats within an experiment replicate in the myrosinase assays. Sample sizes were *N* = 9132 for *P. brassicae* and *N* = 2441 for *P. xylostella* herbivory screens, *N* = 930 for myrosinase activity assays, *N* = 1484 for trichome density, and *N* = 972 for glucosinolates. Again, data from *P. brassicae* herbivory screens were also used to analyze variation in plant diameter.

### QTL Mapping and Analysis

Windows QTL cartographer V2.5 [55] was used for composite interval mapping (CIM) of QTL. The standard model (Model 6) was used with forward regression, a window size of 10 cM, and 5 background control markers. QTL were scanned at a walk speed of 0.5 cM. Statistical significance of QTL for each trait was assessed by permuting each data set 1000 times, with a significance level of 0.05. Furthermore, 2-LOD support intervals [56] were obtained from the QTL cartographer output. For each QTL, the effect strength was estimated as the proportional difference, (LSM_Da(1)-12_−LSM_Ei-2_)/((LSM_Da(1)-12_+LSM_Ei-2_)/2), where LSM is the ANOVA least squares mean at the marker closest to the QTL peak. Positive values indicate that the Da(1)-12 genotype has a stronger effect, negative values that Ei-2 has a stronger effect. Finally, the proportion of explained variance, *R* ^2^, was obtained from the QTL cartographer output.

In addition, Bayesian interval mapping (BIM) [57] as implemented in R/bim (http://www.stat.wisc.edu/∼yandell/qtl/software/bmqtl) was used. For each trait, 400,000 Markov-Chain-Monte-Carlo steps were simulated and iterations were recorded at every 400^th^ step, with 1000 pre-burn-in and 20000 burn-in steps. Prior for the number of QTL was Poisson, with zero initial QTL.

CIM and BIM yielded very similar results, and the high density probability peak from BIM for a given QTL was usually found within the 2-LOD support interval for a QTL in CIM (**Figure 3**). The most notable difference between both methods was a weak QTL for *Plutella* herbivory that was identified with BIM but not with CIM. Since this QTL was located at the same position as one of the QTL for *Pieris* herbivory, we consider this QTL real (**Figure 3**).

Because epistatic interactions appear to be an important factor in the genetic architecture of complex traits [58] and have been documented for glucosinolate biosynthesis [12], the markers most closely linked to QTL peaks were tested for potential epistatic interactions. Based on this *a priori* expectation that main QTL might interact with one another, a significance threshold of 0.05 was chosen. First, for every trait all single markers and all pairwise interactions between these markers were included in the ANOVA models. If more than one significant interaction with a particular marker was detected, also higher-order interaction terms between markers were included.

## Supporting Information

Table S1 Genotype data for Da(1)-12×Ei-2 RILs
(0.26 MB XLS)

Table S2 Genes of the glucosinolate-myrosinase system, AGI numbers, and references
(0.06 MB DOC)
