# Supplementary material for: Mapping of QTL for Resistance against the Crucifer Specialist Herbivore Pieris brassicae in a New Arabidopsis Inbred Line Population, Da(1)-12×Ei-2
Source: PLoS One. 2007 Jun 27;2(6):e578. doi: 10.1371/journal.pone.0000578 (PMC1892800; doi:10.1371/journal.pone.0000578)
Supplement: Table S2 — Genes of the glucosinolate-myrosinase system, AGI numbers, and references (0.06 MB DOC) [file pone.0000578.s002.doc]

**Supporting Table 1:** **Genes of the Glucosinolate-Myrosinase System**

| **Gene** | **AGI number** | **Description** | **References** |
| --- | --- | --- | --- |
| *CYP79F1* | At1g16410 | Cytochrome P450: converts amino acids to aldoximes | 1-4 |
| *CYP79F2* | At1g16400 | Cytochrome P450: converts amino acids to aldoximes | 1-4 |
| *AtST5a* | At1g74100 | Desulfoglucosinolate Sulfotransferase | 5 |
| *AtST5b* | At1g74090 | Desulfoglucosinolate Sulfotransferase | 5 |
| *AtST5c* | At1g18590 | Desulfoglucosinolate Sulfotransferase | 5 |
| *ESP* | At1g54040 | Epithiospecifier protein; determines glucosinolate hydrolysis product identity | 6 |
| *UGT74B1* | At1g24100 | UDP-glucose:thiohydroximate *S*-glucosyltransferase | 7 |
| *C-S lyase* | At2g20610 | Formation of thiohydroximates | 8 |
| *CYP79B2* | At4g39950 | Cytochrome P450: converts amino acids to aldoximes | 9-11 |
| *CYP79B3* | At2g22330 | Cytochrome P450: converts amino acids to aldoximes | 9,11 |
| *IQD1* | At3g09710 | binds to calmodulin in a Ca^2+^-dependent fashion; affects expression of multiple genes with roles in glucosinolate metabolism | 12 |
| *ESM1* | At3g14210 | Epithiospecifier modifier 1; alters glucosinolate hydrolysis; ESM1 represses nitrile formation and favors isothiocyanate production | 13 |
| *BCAT4* | At3g19710 | Branched-chain amino transferase; involved in chain elongation pathway in biosynthesis of Methionine-derived glucosinolates | 14 |
| *AOP1* | At4g03070 | 2-oxoglutarate-dependent dioxygenase | 15 |
| *AOP2* | At4g03060 | 2-oxoglutarate-dependent dioxygenase | 15 |
| *AOP3* | At4g03050 | 2-oxoglutarate-dependent dioxygenase | 15 |
| *CYP83A1* | At4g13770 | Cytochrome P450: converts aldoximes to *S*-alkylthiohydroximates | 16,17 |
| *CYP83B1* | At4g31500 | Cytochrome P450: converts aldoximes to *S*-alkylthiohydroximates | 16,17 |
| *CYP79A2* | At5g05260 | Cytochrome P450: converts amino acids to aldoximes | 18 |
| *MAM1* | At5g23010 | Methylthioalkylmalate synthase; involved in methionine carbon-chain extension | 19-22 |
| *MAM2* |  | Methylthioalkylmalate synthase; involved in methionine carbon-chain extension; not present in Col-0 | 19,20,22 |
| *MAML* | At5g23020 | Methylthioalkylmalate synthase; involved in methionine carbon-chain extension | 19,23 |
| *TGG1* | At5g26000 | Myrosinase: gucosinolate hydrolysis | 24 |
| *TGG2* | At5g25980 | Myrosinase: gucosinolate hydrolysis | 24 |
| *TGG3* | At5g48375 | Myrosinase: gucosinolate hydrolysis, pseudogene in Col-0 | 25 |
| *ATR1* | At5g60890 | Myb-like transcription factor, tryptophan gene regulator | 26 |

SUPPLEMENTARY LITERATURE CITED

1. Hansen CH, Wittstock U, Olsen CE, Hick AJ, Pickett JA et al. (2001) Cytochrome P450 CYP79F1 from Arabidopsis catalyzes the conversion of dihomomethionine and trihomomethionine to the corresponding aldoximes in the biosynthesis of aliphatic glucosinolates. J Biol Chem **276:** 11078–11085

2. Reintanz B, Lehnen M, Reichelt M, Gershenzon J, Kowalczyk M et al. (2001) *bus*, a bushy Arabidopsis *CYP79F1* knockout mutant with abolished synthesis of short-chain aliphatic glucosinolates. Plant Cell **13:** 351–367.

3. Chen S, Glawischnig E, Jorgensen K, Naur P, Jorgensen B et al. (2003), CYP79F1 and CYP79F2 have distinct functions in the biosynthesis of aliphatic glucosinolates in Arabidopsis. Plant J **33:** 923–937

4. Tantikanjana T, Mikkelsen MD, Hussain M, Halkier BA, Sundaresan V (2004) Functional analysis of the tandem-duplicated P450 genes *SPS*/*BUS*/*CYP79F1* and *CYP79F2* in glucosinolate biosynthesis and plant development by Ds transposition-generated double mutants. Plant Physiol **135:** 840–848

5. Piotrowski M, Schemenewitz A, Lopukhina A, Müller A, Janowitz T et al. (2004) Desulfoglucosinolate sulfotransferases from *Arabidopsis thaliana* catalyze the final step in the biosynthesis of the glucosinolate core structure. J Biol Chem **279:** 50717–50725

6. Lambrix V, Reichelt M, Mitchell-Olds T, Kliebenstein DJ, Gershenzon J (2001) The Arabidopsis epithiospecifier protein promotes the hydrolysis of glucosinolates to nitriles and influences *Trichoplusia ni* herbivory. Plant Cell **13:** 2793–2807

7. Grubb CD, Zipp BJ, Ludwig-Müller J, Masuno MN, Molinski TF et al. (2004) Arabidopsis glucosyltransferase UGT74B1 functions in glucosinolate biosynthesis and auxin homeostasis. Plant J **40:** 893–903

8. Mikkelsen MD, Naur P, Halkier BA (2004) Arabidopsis mutants in the *C-S* lyase of glucosinolate biosynthesis establish a critical role for indole-3-acetaldoxime in auxin homeostasis, Plant J **37:** 770–777

9. Mikkelsen MD, Hansen CH, Wittstock U, Halkier BA (2000) Cytochrome P450 CYP79B2 from *Arabidopsis* catalyzes the conversion of tryptophan to indole-3-acetaldoxime, a precursor of indole glucosinolates and indole-3-acetic acid. J Biol Chem **275:** 33712–33717

10. Hull AK, Rekha V, Celenza JL (2000) Arabidopsis cytochrome P450s that catalyze the first step of tryptophan-dependent indole-3-acetic acid biosynthesis. Proc Natl Acad Sci USA **97:** 2379–2384

11. Glawischnig E, Hansen BG, Olsen CE, Halkier BA (2004) Camalexin is synthesized from indole-3-acetaldoxime, a key branching point between primary and secondary metabolism in *Arabidopsis*. Proc Natl Acad Sci USA **101:** 8245–8250

12. Levy M, Wang Q, Kaspi R, Parrella MP, Abel S (2005) Arabidopsis IQD1, a novel calmodulin-binding nuclear protein, stimulates glucosinolate accumulation and plant defense. Plant J **43:** 79-96

13. Zhang Z, Ober JA, Kliebenstein DJ (2006) The gene controlling the quantitative trait locus EPITHIOSPECIFIER MODIFIER1 alters glucosinolate hydrolysis and insect resistance in *Arabidopsis*. Plant Cell **18:** 1524-1536

14. Schuster J, Knill T, Reichelt M, Gershenzon J, Binder S (2006) BRANCHED-CHAIN AMINOTRANSFERASE4 is part of the chain elongation pathway in the biosynthesis of methionine-derived glucosinolates in *Arabidopsis*. Plant Cell **18:** 2664-2679

15. Kliebenstein DJ, Lambrix VM, Reichelt M, Gershenzon J, Mitchell-Olds T (2001) Gene duplication and the diversification of secondary metabolism: side chain modification of glucosinolates in *Arabidopsis thaliana.* Plant Cell **13:** 681-693

16. Hemm MR, Ruegger MO, Chapple C (2003) The Arabidopsis *ref2* mutant is defective in the gene encoding CYP83A1 and shows both phenylpropanoid and glucosinolate phenotypes. Plant Cell **15:** 179–194

17. Naur P, Petersen BL, Mikkelsen MD, Bak S, Rasmussen H et al. (2003) CYP83A1 and CYP83B1, two nonredundant cytochrome P450 enzymes metabolizing oximes in the biosynthesis of glucosinolates in Arabidopsis. Plant Physiol **133:** 63–72

18. Wittstock U, Halkier BA (2000) Cytochrome P450 CYP79A2 from *Arabidopsis thaliana* L. catalyzes the conversion of L-phenylalanine to phenylacetaldoxime in the biosynthesis of benzylglucosinolate. J Biol Chem **275:** 14659–14666

19. Kroymann J, Textor S, Tokuhisa JG, Falk KL, Bartram S et al. (2001) A gene controlling variation in *Arabidopsis thaliana* glucosinolate composition is part of the methionine chain elongation pathway. Plant Physiol **127:** 1077–1088

20. Kroymann J, Donnerhacke S, Schnabelrauch D, Mitchell-Olds T (2003) Evolutionary dynamics of an Arabidopsis insect resistance QTL. Proc Natl Acad Sci USA **100:** 14587–14592

21. Textor S, Bartram S, Kroymann J, Falk KL, Hick A et al. (2004) Biosynthesis of methionine-derived glucosinolates in *Arabidopsis thaliana*: recombinant expression and characterization of methylthioalkylmalate synthase, the condensing enzyme of the chain elongation cycle. Planta **218:** 1026–1035

22. Benderoth M, Textor S, Windsor AJ, Mitchell-Olds T, Gershenzon J et al. (2006) Positive selection driving diversification in plant secondary metabolism. Proc Natl Acad Sci USA **103:** 9118-9123

23. Field B, Cardon G, Traka M, Botterman J, Vancanneyt G et al. (2004) Glucosinolate and amino acid biosynthesis in Arabidopsis. Plant Physiol **235:** 828-839

24. Rask L, Andréasson E, Ekbom B, Eriksson S, Pontoppidan B et al. (2000) Myrosinase: gene family evolution and herbivore defense in Brassicaceae. Plant Mol Biol **42:** 93–113

25. Zhang J, Pontoppidan B, Xue J, Rask L, Meijer J (2002) The third myrosinase gene TGG3 in *Arabidopsis thaliana* is a pseudogene specifically expressed in stamen and petal. Physiol Plant **115:** 25–34

26. Bender J, Fink GR (1998) A Myb homologue, ATR1, activates tryptophan gene expression in *Arabidopsis*. Proc Natl Acad Sci USA **95:** 5655-5660
